# De Quervain’s tenosynovitis: a non-randomized two-armed study comparing ultrasound-guided steroid injection with surgical release

**DOI:** 10.1007/s12306-022-00735-0

**Published:** 2022-02-23

**Authors:** A. K. Bhat, R. Vyas, A. M. Acharya, K. V. Rajagopal

**Affiliations:** 1 Division of Hand and Microsurgery, Department of Orthopaedics, Kasturba Medical College, Manipal Academy of Higher Education, Manipal, 576104 India; 2 Department of Radiodiagnosis, Kasturba Medical College, Manipal Academy of Higher Education, Manipal, 576104, India

**Keywords:** De Quervain’s tenosynovitis, Steroid injection, Ultrasonography, First extensor compartment, Surgical release

## Abstract

**Purpose:**

Ultrasonography is currently used for both diagnostic and therapeutic purposes in de Quervain’s tenosynovitis. There is a dearth of information on how effective an ultrasound-guided (USG) steroid injection is when compared to surgical release of the first extensor compartment. Hence, we performed a non-randomized two-armed comparison study to test our hypothesis that USG guided steroid injection is equally effective as surgery.

**Method:**

62 consecutive patients participated in the study with 32 of them selecting the option of USG guided injection (Set A), and the rest undergoing surgical release (Set B). We reviewed them after 3 and 6 weeks and 6 months for functional outcome using DASH, PRWE and VAS scores, recurrence, or any complications. They were further followed if they were symptomatic.

**Results:**

The DASH/PRWE/VAS scores improved at the end of 6 months from 81.7/79.3/6.8 to 1.0/1.7/1.0, respectively for patients undergoing USG guided steroid injection. Similarly, for the patient undergoing surgery, the scores improved from 82.2/81.5/6.7 to 1.7/3.4/1.0, respectively. This was statistically significant in both the groups (*p* < 0.05) and was comparable to each other. Two patients in Set A came back with recurrence at eight and 10 months and two reported occasional pain on heavy work. Three patients had tenderness and two had numbness in Set B at the scar site.

**Conclusion:**

We observed that USG guided steroid injections are comparable to surgical release in terms of pain relief, functional outcome, complications.

## Introduction

De Quervain’s tenosynovitis (DQV) is primarily a painful tendinosis where tenderness and soreness are felt at the region of the radial styloid while performing activities, which involve wrist and thumb movements [1]. It occurs due to repetitive activities, common in women in the age group of 30–50 years [1–4]. Some authors attribute the condition to be due to inflammatory involvement of the synovial sheath inside the first dorsal compartment (FDC) which surrounds the abductor pollicis longus (APL) and the extensor pollicis brevis (EPB) tendons. Samples collected from affected patients have shown the presence of neutrophil elastase and cyclooxygenase which indicates the presence of inflammation and hence the damaging effects on the collagen structure in the FDC [5]. However, other studies have objected to the term ‘tenosynovitis’ in DQV, as no inflammation could be demonstrated. The tendinosis is more of myxoid degeneration rather than acute inflammation that is induced due to mechanical impingement of the Abductor pollicis longus (APL) and extensor pollicis brevis (EPB) tendons in the tight fibro-osseous canal, which stimulates the nociceptors [1–4]. The thickening of the synovial sheath may result in entrapment of the tendon in the tight canal during an excursion of the APL and EPB tendons [1–5]. There is increased friction between the contents of the canal, which could be the source of pain and tenderness. [1–6]. Anatomical variations in the first extensor compartment such as the presence of a separate septum for EPL and multiple slips of APL are also said to be associated with De Quervain’s tenosynovitis [5–7].

Patients undergo a spectrum of treatment including non-surgical measures of rest, splinting, casting, non-steroidal anti-inflammatory drugs (NSAIDs), and local steroid injections [1]. If the above measures fail, surgical treatment remains the only option [1]. However, failure of treatment and recurrence have been described in both non-operative and surgical modalities due to missed or incomplete release of sub-compartment for EPB in the majority of studies [1, 3, 5–9].

Non-surgical management other than steroids has shown limited benefits and a high rate of relapse [1, 6, 10, 11]. Steroid injections are a widely accepted and its efficacy has been proved in randomized control trials (RCT) and systematic reviews [1, 4, 12, 13]. However, with blind injections, the recurrence rates from the literature have varied from nil to as high as 52% [1]. This is attributed to be due to septation of the first extensor compartment where the blind injections can miss one of the sub-compartments and lead to inadequate relief of symptoms as shown in cadaveric and clinical studies [14, 15]. The use of steroids has also shown complications like injury and rupture of APL, EPB and dorsal sensory branches of radial nerve apart from flare, hypopigmentation and atrophy of the skin [16–18]. The introduction of ultrasonography (USG) guided steroid injections has brought in a new paradigm as it can visualize the presence of a sub-compartment and allow for more accurate placement of the needle for steroid injection [19, 20]. The success of USG guided steroid injections as shown in some of the reports in the last decade introduces it as a potential primary mode of treatment for de Quervain’s tenosynovitis [19–26]. However, it remains to be seen whether it can produce an equally better result when compared to a carefully performed surgical release where excellent and predictable long-term results have been reported [27, 28]. There are no studies comparing these two modalities of treatment. According to our hypothesis, both treatments are comparable for the treatment of de Quervain’s tenosynovitis. Associated complications of surgery can be avoided if USG guided steroid injection was found to have equal outcomes.

## Materials and methods

This was a non-randomized two-arm study conducted after institutional ethical board clearance. The aim of the study was to compare the functional outcome, recurrence and complications comparing USG guided steroid injection with surgical release of the first extensor compartment. Consecutive patients with clinically diagnosed de Quervain’s tenosynovitis were selected as defined by radial sided wrist pain, radial-styloid tenderness, a positive Finkelstein’s test, and those who did not improve after a trial of conservative management of a minimum of 4 weeks were included in the study. Patients who had taken prior treatment with multiple steroid injections, those who had a contraindication to steroid therapy, those with causes like past fractures/dislocations, inflammatory disorders or previous surgery at the same region, CMC or wrist arthritis or neoplasm which would have altered the functional outcomes were excluded (Table 1).

**Table 1:** Demographic data of the two sets of patients

Parameter		Patient details	
		USG guided injection	Surgical release
Gender	Female/total	26/32	21/30
Mean age	In years	46.17	49.13
Occupation: number	Labourer	3	4
	Homemaker	20	20
	Student	2	0
	Desk job	6	4
	Nurse	1	2
Dominant hand involvement		20/32	18/30
Right hand involvement	Right hand/total	23/32	22/30
*Duration of symptoms*			
Mean (for total patients)			
23 weeks( 4–80 weeks)	Upto 3 months	17	14
	Upto 6 months	9	13
	Upto 9 months	2	2
	Upto 12 months	4	1
Patients with comorbidities		4	8
Patients with history of injury	Injury/total	7/32	6/30
Separate compartment for EPB	Septum/total	18/32	16/30
Multipennate APL	Multipennate/total	29/32	19/30

62 consecutive patients were grouped into Set A (USG guided injection—32) and Set B (Surgical release—30). The demographic characteristics of these two groups were comparable. (Table 1). Patients were allotted based on shared decision-making of their choices after explaining both the procedures in detail and discussion with the operating surgeon. All patients underwent pre-operative USG examination to aid in confirming the diagnosis of tenosynovitis, and more importantly, to note the presence of anatomical variations. This information would aid both the operating surgeon and the radiologist during their intervention procedures. Qualified radiologists from the department of radiology with more than 5 years of experience in musculoskeletal ultrasonography did the USG examinations.

**Fig. 1:**
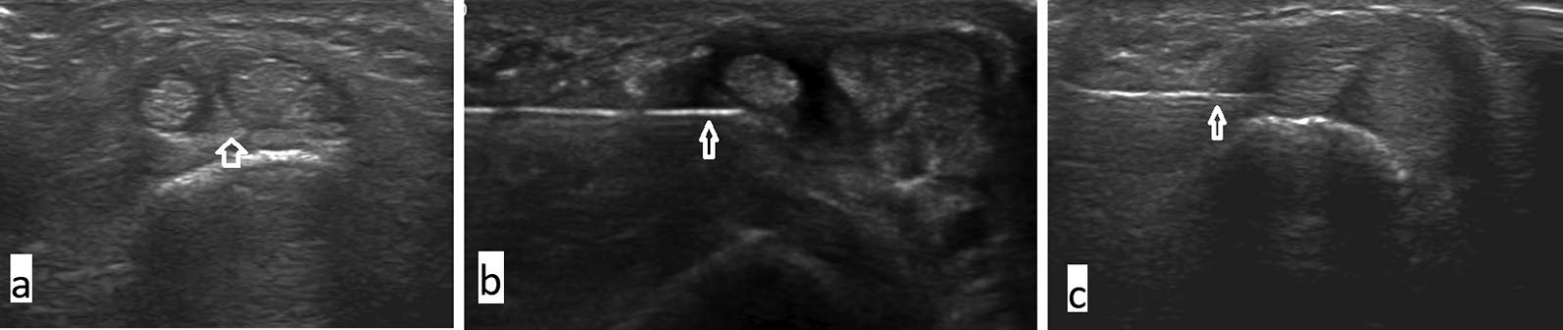
**a** USG images with arrow showing the septum in the first dorsal compartment. **b** Arrow showing the needle in the EPB compartment with infiltrating. Note that the infiltrate has not reached the APL compartment due to the presence of a septum in between. **c** Arrow showing the needle in the first dorsal compartment. Note that the infiltrate has evenly spread around both the tendons

A pilot study was done initially in six patients to standardize the technique of USG guided injection. Transverse and longitudinal approaches were used, and the former was found to be better, as it was easy to access both the compartments and avoid the sensory branches of the radial nerve (Fig. 1). The injections were given by a single dedicated musculoskeletal radiologist (KVR) with more than 15 years experience to ensure the uniformity of technique.

### Injection technique

Under aseptic conditions, a Philips IU 22 USG machine and 12.3 Hz linear probe were used to visualize cross-sectional anatomy of the first extensor compartment. A 20-gauge needle was used to inject 2 cc of 2% lignocaine and 20 mg methylprednisolone acetate (20 mg/ml) into the first extensor compartment of the wrist around the APL and EPB tendons.

If a separate compartment was noted for EPB, the injection was split equally for both the compartments with similar infiltration techniques under USG guidance (Fig. 1b). Special care was taken to avoid the sensory branches of the radial nerve. Patients undergoing injections were advised a thumb spica splint and light work for 3 weeks followed by resumption of their routine activities.

### Surgical technique

A single-hand surgeon performed all surgical releases. A transverse incision was made and decompression of the first extensor compartment was completed, ensuring the release of any sub-compartment and checking the excursion of EPB and APL separately. They were immobilized in a thumb spica slab for 3 days and later mobilized in a thumb spica splint for a further 3 weeks. This was followed by the resumption of their activities.

We reviewed all patients after 3 and 6 weeks and 6 months for the functional outcome, recurrence, or any complications. We used the disabilities of arm, shoulder and hand (DASH) score, patient rated wrist evaluation (PRWE) score, and visual analogue score (VAS) [13, 16]. The 30-item self-reported DASH questionnaire looks at the patient’s ability to perform specific upper extremity activities and difficulty and interference with daily life. The score is documented on a 5-point Likert scale. PRWE is a 15-item questionnaire used for specific wrist problems and was developed to assess wrist pain and disability in activities of daily living. It has two subscales of pain and function and each of them has an item (five for pain and 10 for function) rated from 1 to 10. The maximum score for each of these two sections is 50 and the minimum is 0. The VAS is a widely used unidimensional measure of pain intensity. We followed a simple method in using a straight horizontal line of 10 cm orientated from the left (worst – maximum pain at 10 cm mark) to the right (best – no pain at zero mark). The patient marks the point on the line, which they perceive to be their current state of pain at the site of pathology. The lesser the score suggests a better outcome in all the three scores.

We chose 6 months as this was the maximum likely time gap after which recurrence was unlikely to be reported [10, 29]. However, patients who returned back after 6 months were also assessed based on their symptoms and signs and their status documented. Patients who did not improve after the single steroid injection and had a relapse of symptoms was planned to be treated surgically. Relapse rate and intraoperative findings were to be recorded to establish etiology.

### Statistical methodology

Mean scores were calculated for DASH, PRWE and VAS scores at every visit and were compared using repeated ANOVA measures. Total change in percentage was used to compare scores at each follow-up. Relapse rates and changes in treatment were reported. *P*-value < 0.05 was considered statistically significant.

## Results

Table 1 shows the demographic characteristics of both sets of patients. The majority of them were females and homemakers in their 4th decade. Among the 62 patients, 82.2% were middle-aged women with a mean age of 46.1 years involved in household activities. Most of our patients were right-handed (72%) having symptoms on the dominant hand (61%). (Table 1) The mean duration of symptoms was 23 weeks (4–80 weeks). About 80% of the patients did not have any associated comorbidities. On pre-op USG evaluation, 48 patients (77.4%) had multipennate APL, 34 patients (54.8%) had sub-compartment for EPB and one patient was found to have absent EPB (Hiranuma type 4) [21].

### Functional outcome

The mean DASH, PRWE and VAS scores of patients in both the groups improved marginally following the initial conservative management of 4 weeks, which was found to be insignificant (Table 2).

**Table 2:** Figure showing the VAS, PRWE and VAS scores for patients undergoing surgical release and steroid injections

Follow up	DASH		PRWE		VAS	
	Surgical release	Steroid injection	Surgical Release	Steroid injection	Surgical release	Steroid injection
On first consultation	82.2 ± 11.4	81.7 ± 9.5	81.5 ± 6.2	79.3 ± 7.9	6.7 ± 1.2	6.8 ± 1.2
3 weeks (conservative Mx)	76.7 ± 12.15	73.2 ± 14.7	79.6 ± 9.0	75.1 ± 10.9	4.9 ± 1.8	4.8 ± 1.6
6 weeks	5.5 ± 2.7	6.6 ± 2.7	11.9 ± 5.4	9.1 ± 6.5	2.2 ± 1.1	2.6 ± 1.3
6 months	1.7 ± 1.9 (*P* < 0.05)	1.0 ± 1.6 (*P* < 0.05)	3.4 ± 2.6 (*P* < 0.05)	1.7 ± 2.1 (*P* < 0.05)	1.0 ± .0 (*P* < 0.05)	1.0 ± .0 (*P* < 0.05)

At 6 weeks, both the group of patients showed significant improvement (Table 2). The DASH/PRWE/VAS scores improved from 82.2/81.5/6.7 to 1.7/3.4/1.0, respectively for patients undergoing surgery (Table 2). Similarly, for a patient undergoing USG guided steroid injection, the scores improved from 81.7/79.3/6.8 to 1.0/1.7/1.0, respectively (Table 2). At the end of 6 months, there was a statistically significant difference in both the groups (*p* < 0.05) and were comparable to each other.

### Recurrence

We did not observe recurrence in either group at the end of 6 months of follow-up. Twelve patients came for follow-up after 6 months out of which seven had received USG guided steroid injections. Those patients had an average follow-up of 14.5 months (12–24 months). Two patients who had received USG guided steroid injections showed recurrence, which was observed at eight and 10 months, respectively after the index injection. Both of them incidentally had a single compartment for EPB tendon on USG evaluation. One of them was a 35-years-old computer software professional who presented 8 months after the injection. She had symptoms for 4 weeks prior to the presentation. Another patient was a 59-year-old homemaker who had a history of soft tissue trauma involving the wrist 8 months after the index injection. She presented to us 2 months later with symptoms of recurrence. Both patients were unwilling for surgery and hence continued on medications and splinting.

### Complications

Apart from two patients with recurrence, two other patients reported pain only on heavy work and the rest had complete relief of symptoms and returned to their daily activities.

Out of those 30 patients who underwent surgical release, three had scar tenderness, which resolved by the 6th month follow-up. Two patients have decreased sensations over the surgical scar and the rest had complete relief of symptoms.

## Discussion

### Relevance and importance of ultrasound for steroid injections

Corticosteroid injection is a popular option for musculoskeletal disorders ever since their introduction in the 1950s [4]. RCTs and systematic review of RCTs in the last two decades have consistently shown their benefit in de Quervain’s tenosynovitis [1, 4, 12, 13]. Rowland et al. did a systematic review, which included Five RCTs with 142 patients receiving corticosteroid injection with an overall follow-up period of 5.6 months (1.5–12 months) [13]. They showed resolution rates of symptoms higher than the controls (2.59 vs. 5.37, (*p* = 0.05)). The steroid group demonstrated better pain relief as measured by the visual analogue scale (VAS) and improvement in Dutch AIMS-HFF and DASH scores that were statistically significant [13]. Our study confirms the benefit observed with steroids but we additionally used the advantage of an ultrasound-guided procedure. We conducted this study to test our hypothesis that USG guided steroid injection is equally effective as surgery. We observed that USG guided steroid injections are comparable to surgical release in terms of pain relief, functional outcome, complications.

Blind steroid injection has a high chance of missing the separate compartment for EPB and hence leading to relapse or incomplete relief. Ultrasound has been used as a key tool for the diagnosis of de Quervain’s tenosynovitis and more importantly for subtle anatomical variations like separate compartments for APL and EPB. Additionally, its use to deliver steroids when compared to blind injection has significantly improved outcomes as shown in some of the reports [21–26] (Table 3). In a series of 50 cadavers, Leversedge et al. showed an accuracy rate of 94% in the diagnosing presence of sub-compartments within the first compartment using USG. Their USG -guided injection accuracy was 100% and EPB compartment injection 96% [24]. Another study, by Kang et al. in a series of 15 fresh cadavers, demonstrated not only a higher accuracy for USG guided injections (93.3% vs. 40%) to target the first extensor compartment but also better results when separate septum was present (85.7% vs. 16.7%) [30]. In a cadaver model involving 43 specimens comparing USG and anatomical landmark (AL) guided injection, Kutsikovich et al. showed that both techniques had an infiltration rate of 100% [32]. In the presence of a septum, the USG group achieved a higher infiltration rate in EPB sub-compartments of 75% accuracy in reaching the sub-compartment when compared to 33% in AL. Although statistically unconfirmed, they stated that USG aided injections in the presence of a septum [32]. In our series, more than 50% of patients had a separate sub-compartment and none of them came back with recurrence of symptoms, suggesting the effectiveness of USG guided injections.

**Table 3:** Figure showing some of the key articles on ultrasound based cadaveric and clincal studies and surgical decompression

Authors	Year	No. of cadavers	Study design	Results (Was USG more accurate?)	Key observations
*Ultrasound based cadaveric studies*					
Leversedge et al. [24]	2016	50	To evaluate the accuracy in regard to:1) anatomic	-USG evaluation accuracy was **higher**—94% assessment, 2) needle placement without imaging guidance: 3) ultrasonography-guided injection with priority for the extensor pollicis brevis sub-compartment -When ultrasonography was not used: Accurate needle placement—26/50 wrists (52%), and only 2 of 27 needles (7%) were located within the EPB sub-compartment -Ultrasonography-guided injection—100% accurate (50/50) and EPB injection was 96% accurate (26 of 27) when two compartments were present	A sub-compartment identified in 27/50 wrists; 18 complete and 9 incomplete Minimal extravasation was identified in 6 of 50 wrists (12%)
Kang et al. [30]	2017	15	To evaluate the accuracy of USG injection aimed at EPB tendon sheath and anatomical variances influencing the results. Comparison between manual and USG injections in 30 cadaveric wrists	The accuracy was **higher** in the USG group (93.3%) than in control group (40.0%) The accuracy in manual group without septum (55.6%) was higher than in those with septum. (16.7%). The accuracy between those without septum (100%) and with septum (85.7%) was not significantly different in USG group	Tendency not to have septum was seen in wrists with more EPB or APL tendon slips. All intratendinous injections was occurred in the wrist with 1 EPB tendon slip or 1 or 2 APL tendon slip
Kutsikovich et al. [32]	2018	43	To determine the accuracy of USG injections in comparison with anatomical landmark (AL) based injections. Hypothesis: Accuracy of injection would be increased by ultrasound guidance Randomised study of cadaveric specimens to receive latex dye injections either by USG or using AL technique in the 1^st^ dorsal compartment (FDC)	All specimens demonstrated dye within the FDC. Ultrasound identified septated FDC in 6/8 (sensitivity of 75% and specificity of 92%) versus 2/6 in the AL group demonstrating dye infiltration around the EPB. USG injections had a **higher** observed infiltration rate in EPB sub-compartments than the AL technique. It may help in needle placement in septated FDC Dye noted in the subcutaneous tissues in 2/21 specimens in the USG group vs 2/22 in the AL group	8/21 specimens in the UG group and 6/22 AL group demonstrated a septated 1^st^ dorsal compartment

Authors	Year	No. of patients	Follow up	Study design	Results	Complications
*Ultrasound based clinical studies*						
Kume.et al. [19]	2012	44	4 weeks	Randomly allocated USG guided and manual injection. Pain evaluation by VAS score at 4 weeks	US-guided injection showed a more significant decrease in VAS score than the manual injection group. two in USG group and nine in manual group underwent surgery at 6 weeks	No adverse events till 6 weeks
McDermott et al. [23]	2012	40	11 months	A prospective consecutive series of patients referred for an ultrasound-guided injection. DASH and VAS scores assessed at follow up	At the 6-week 97% had at least partial resolution of symptoms. Multiple sub-compartments were identified in 52% of cases. No adverse effects were noted	14% of wrists had recurrence and all of them had sub-compartments on ultrasound
Hajder et al. [22]	2013	62	12.4 months	Retrospective case series of patients who were evaluated after USG guided steroid injection	After two injections, 91% of the patients had good long-term results,	local depigmentation, allergic reaction, paresthesia of the superficial branch of radial nerve
Keating-Hart et al. [31]	2016	41	15.6 months	Prospective, open-label study to find clinical and ultrasound features which could predict the failure of conservative treatment in de Quervain’s syndrome. Ultrasound-guided injection performed in 41 patients	Patients with positive clinical tests, high VAS score and presence of intra-compartmental septum, had a significantly higher risk of failure following conservative treatment	Recurrence in 10/41
Bing et al. [25]	2018	28	31.7 days	Different steroid USG injection techniques based on anatomical variations of presence of septum of the first extensor compartment and to evaluate its usefulness in patients	22 out of 23 patients were satisfied with the results with significant drop in pain scores	mild depigmentation noted at the injection site

Authors	Year	No. of Patients	Follow up	Risks identified	Functional results	
*Results of surgical decompression*						
Scheller et al. [27]	2009	94	15.7 years	None reported	All patients had complete relief of symptoms and returned to their normal daily activities. No cases of recurrence	Superficial wound infection, delayed wound healing, transient sensory radial nerve lesion
Lee et al. [35]	2014	33	28.4 months	18/33 (55%), had separate EPB compartment, A thick retinaculum and neovascularization of the tendon sheath were noted. Small ganglia from the tendon sheaths noted in eight patients	All patients except one improved. average VAS score decreased from 7.42 to 1.33 and DASH from 53.2 to 3.45 postoperatively	One had negative sign on Finkelstein test at the last follow-up There were no postoperative complications
Garcon et al. [28]	2018	89	9.5 years	A supernumerary septum in 50 cases (56%), and APL tendon multiple slips in 35 cases	There were no recurrences. Functional impairment was absent in 68 wrists, Mean VAS was 0.76	Functional impairment moderate in 8 and significant in 4, dissatisfaction in associated disease cases
Mangukiya et al. [34]	2019	46	3 months	Severe peritendinous adhesions, ganglion within first dorsal compartment	Mean DASH score improved from 42.26 to 5.37, Mean VAS from 7.30 to 2.33 post-operatively	hypertrophic scar, persistent numbness to first dorsal web space due to injury to superficial radial nerve, recurrent symptoms
Salim et al. [36]	2021	40	6 months	Not reported	All patients improved after surgery. (VAS score, Quick DASH Score, and Mayo Wrist Score improved)	tendon subluxation

Similar results were observed in clinical studies [18, 20, 22, 24]. In a series of 43 wrists who were evaluated with USG, Kwon et al. showed a sensitivity of 100% and a specificity of 96% with a positive predictive value of 95% in diagnosing an intra-compartmental septum [20]. Kume et al. did an RCT involving 44 wrists comparing blind injections with USG guided injections [18]. At 4 weeks, they reported a significant decrease in the VAS scores in the USG group from 80.3 to 25.6 mm(*p* = 0.004) when compared to the manual group (from 78.0 to 58.2 mm(*p* = 0.04)) after injection [19]. Their results suggested that USG guided injections targeted the septation of EPB better than the blind injections [19]. However, there have been reports which have questioned the advantage of the use of USG including those with reports of increased chance of failure particularly with the presence of a sub-compartment [16, 23, 31, 32]. Nevertheless, its benefit and use are still advocated in view of a higher chance of placement of the needle at the pathological site [23, 31, 32]. In a level 1, study of randomized comparison between blind and USG guided injection involving 44 patients, Shin et al. observed sustained improvement in PRWE and pain scores at 4 and 12 weeks post-injection [16]. The authors observed that at 3 months, 10% in USG showed recurrence of symptoms, which was marginally better than 26.3% in the blind group although this was statistically not significant [16]. In our study, we observed a benefit of USG-guided steroid injection after 6 months of follow up which was statistically significant. Sustained benefit of injections was seen in 93.7% of our patients. None of the patients with higher risks for recurrence, came back with relapse of symptoms. The major advantage of a USG guided procedure lies in its ability to diagnose the condition, checking for anatomical variability, while helping in counselling the patient on the prognosis of the condition [22]. Additionally, it helps in the accurate placement of the needle at the right pathological site [22]. The procedure is relatively inexpensive in our country costing $15 in our institution and hardly takes a little more time than blind injections, which incidentally costs $10. In the two patients with recurrence that was observed in our series after 6 months, we observed a common compartment for APL and EPB. In one of the patients who was a computer professional, we were unable to find the exact etiology. In the other, a homemaker, the soft tissue injury may have independently triggered a new episode. Incidentally, no studies have established an association between hand usage at work or trauma with this condition [33].

### Functional outcomes

The literature continues to report complications associated with surgery like wound infections, delayed wound healing, radial nerve injury, scar hypertrophy and tenderness [27, 34]. In this regard, the role of USG guided steroid injections becomes important as recent reports suggest equally impressive results with them as well [19, 21–26]. Hence we aimed to address the question of whether USG guided injections offers a success rate comparable to surgery apart from its obvious advantage of saving time, cost and logistics for both patients, surgeons and insurance. There are no studies on comparison between the above two extremely effective procedures. Our study fills this information gap with a unique study comparing the two with respect to functional outcomes, recurrence and complications over a period of 6 months follow-up with a further assessment for recurrence for a maximum of 2 years.

Surgical release of the first extensor compartment has been the gold standard for de Quervain’s tenosynovitis [34]. Long-term follow-up studies have shown impressive results with no reports of recurrence [27, 28] (Table 3). In a series involving 94 patients undergoing surgical release with an average follow-up of 15.7 years, Scheller et al. achieved complete relief of symptoms and normal return to daily activities in all their patients [27]. Garcon et al. also reported no recurrence and a satisfaction rate of 97.5% in a series of 89 patients with follow-up of 9.5 years [28]. Our results of surgery confirm similar outcomes with no recurrences and improvements in functional outcome scores that were statistically significant. We noted that patients who underwent surgical release for de Quervain’s tenosynovitis had significant relief even on the first follow-up after surgery, and the same was maintained until the final follow-up. The total percentage change in DASH scores, PRWE scores and VAS scores at 6 months was also significant. This suggests that surgery is an effective treatment for de Quervain’s tenosynovitis.

However, in our study, USG guided steroid injection provided equally rewarding results as surgery did. We observed significant and sustained relief in the patients treated with USG guided steroid injection and the results are comparable with other studies done for the same [16, 19, 21–29, 29, 30, 30, 31, 31, 32, 32–39]. Steroid injections when given under USG guidance can yield results, which are as good as surgery and avoid complications related to surgery and blind injections.

### Recurrence and complications

Most of the studies noted good relief in the first 4–6 weeks of follow-up after which relapse of symptoms may be observed [10, 11, 13, 14, 16, 23, 31, 37]. However, the pattern of relief and the time of recurrence with the need for a second injection remains variable [9]. Majority of the studies have reported recurrence by 6–12 weeks [9, 14, 18, 20, 30, 37] (Table 3). Earp et al. have reported relief in more than half among a series of 50 consecutive patients who were followed up over a year after a single-blind steroid injection for de Quervain’s tenosynovitis [29]. In their opinion, recurrence wase observed within the first 6 months of follow-up [29]. These recurrences are significantly lower at 6 months whenever USG guided injections were used as nearly 3/4th of patients showed significant relief in other series [22, 23, 29, 31]. Even with USG guided injections, recurrences were observed more frequently in the presence of a separate septum [22, 31]. In one report, Hajdar et al. achieved 73% success after single USG guided injections with all recurrences being observed by 9 months similar to that of our patients [22]. Similarly, Keating-Hart et al. achieved 91% success initially, which was sustained at 76% at the end of the year with five patients each showing recurrence in the initial 6 weeks and later half, respectively [31]. Considering this fact, USG becomes an advantageous tool to counsel our patients on the prognosis of the condition while giving the injections. None of our patients required the need for a surgery even after 1 year of follow-up except two, which justifies the use of USG guided injections ahead of surgery. Literature has noted problems of hypopigmentation, flare reaction, radial nerve symptoms, thrombophlebitis, and weakness of grip strength with steroid injections [1, 10, 16] (Table 3). We did not observe any such adverse reactions in our study.

Incidentally, our patients did not have any significant complications in surgery also, other than minor sensory disturbances at the affected site.

The strength of our study lies in achieving adequate matching of demographics from a similar pool of population while pursuing treatment during the same time period. We have used the DASH, PRWE and VAS scores for the outcome analysis as each of these scores have been shown to correlate with each other well and has also been used for assessment of function following both surgery and steroid injections in past studies [16, 38].

Limitations of our study lie in our inability to provide accuracy rates of diagnostic USG for comparison with therapeutic procedure findings as the injections, imaging evaluations and surgery were all done by different consultants. Although it was not the aim, functional scores at 1-year follow-up would have added value to our study. As expected, most of our patients’ visits were untimely and irregular after 6 months, which is the case in most clinical conditions where patients get optimum relief. Hence, other than features of recurrence, functional scores would not have been useful for comparison. We have not done a detailed evaluation after 6 months, as this was not the aim of the study and have only evaluated for recurrence due to which we were unable to provide long-term follow-up results. However, recurrences are far much lesser after 6 months as reported in literature [29, 37].

Based on our observations, perhaps future studies could involve comparing the role of site and type of needle placement based on USG findings with various combinations of steroids along with hyaluronic acid preparations [25, 26]. The advent of WALANT technique for de Quervain’s and its benefits in recent RCTs opens up its utility for comparison with USG guided techniques as well [39].

## Conclusion

We observed that USG guided steroid injections are comparable to surgical release in terms of pain relief and functional outcome and with far less cost, complications and hospital stay. Surgical release is the gold standard treatment of de Quervain’s tenosynovitis at present and we suggest that USG guided steroid injection can equal surgical results. The ultrasound findings preoperatively also help in surgical planning in correlating the number of APL tendons and separate compartment of EPB which prepares the surgeon to plan well both for injections and counseling of patients for prognostication.
